# Parameter Design and Performance Evaluation of a Large-Swath and High-Resolution Space Camera

**DOI:** 10.3390/s21124106

**Published:** 2021-06-15

**Authors:** Yingying Sun, Peng Rao, Tingliang Hu

**Affiliations:** 1 Shanghai Institute of Technical Physics, Chinese Academy of Sciences, Shanghai 200083, China; sunyy@mail.ustc.edu.cn (Y.S.); hutingliang@mail.sitp.ac.cn (T.H.); 2 Key Laboratory of Intelligent Infrared Perception, Chinese Academy of Sciences, Shanghai 200083, China; 3 School of Physical Sciences, University of Chinese Academy of Sciences, Beijing 100049, China

**Keywords:** large swath and high resolution, earth observation, rotary-scan, parameter design

## Abstract

A rotary-scan space camera with an area sensor can achieve large width and high-resolution imaging. Designing system parameters properly is important for the application of the rotary-scan space camera. We model the swath, resolution, and overlap rate between frames for such a camera. An optimum algorithm combining the linear weighting method and the Monte Carlo method for system parameter design is proposed based on the model. Then, the performance of the designed system is evaluated using the grid point method. The designed systems can achieve swaths of more than 1000 km and less than 1 m resolution without leakage during the imaging. In the evaluation, the designed system can cover 82.13% of the observation region at the height of 500 km in 6.5 min, and the average repeated observation frequency is 3.26 times per 118 s. The design method is simple and effective in the initial design of the rotary-scan space camera’s system parameters. The system designed can provide “no-leakage and wide coverage by quick scan” and “high-frequency repeated observation over a long visibility period.” This will greatly improve earth observation ability in wide-area search and rescue missions.

## 1. Introduction

Achieving wide-swath and high-resolution imaging have always been an important goal for remote-sensing satellites in earth observation tasks. Traditional space cameras provide high-resolution images with a narrow field of view or low-resolution wide-field images [1]. At present, the general imaging methods used for increasing the imaging swath of optical remote sensing cameras include multi-star networking [2], rapid satellite maneuvering [3], multi-camera combination [4], multi-CCD detector stitching [5], and scanning imaging [6]. Although the first four schemes increase the imaging width to some extent, they also greatly increase the system’s volume, weight, and power consumption, resulting in huge hardware costs. Scanning imaging is a method commonly used for increasing the swath.

The existing scanning modes include push broom, whisk broom, and rotary-scan imaging modes [7]. The former two have been applied in real sensors and expand the swath greatly. For example, SPOT-5 HRG sensors achieve a swath of 60 km and spatial resolution of 4 m at an orbital height of 822 km using push-broom sensors [8]. WorldView-3 sensors can achieve swaths of 65 km and resolutions of 0.31 m at an orbital height of 617 km through push broom and band splicing [9]. The MODIS-T uses a whisk broom to achieve a swath of 1780 km with a spatial resolution of 250 m from an orbital altitude of 824 km [10]. As for the rotary scan mode, the DSP satellite swath is wide enough to cover the entire Earth by adapting the rotary scan mode [11]. For radar and laser sensors, similar circular synthetic aperture radar [12] and Palmer scanning systems [13] project laser pulses on the ground to form a rotary-scan path. At present, rotary-scan imaging mode is not yet applied to spaceborne plane array optical remote-sensing systems.

When compared with the push broom, the rotary-scan camera can expand the swath greatly. Compared with the whisk broom, the rotary scan can provide a more stable scan as its moving mechanism is continuous while the whisk broom moving mechanism is intermittent. Suppose this form of rotary-scan imaging is applied to an LEO array optical remote sensing system. In that case, it is expected to provide coverage of unprecedented area size and high-resolution monitor, which will help pinpoint small events in large areas with sufficient time to respond and improve monitor ability [14]. Thus, this rotary-scan space camera will greatly improve wide-area surveillance ability.

However, there are few studies on the system parameter design of the planar array rotating scanning space camera. The existing relevant literature [15,16] only makes a simple analysis of the parameters of the planar array rotating scanning imaging system and does not propose a streamlined design method. Therefore, a more accurate system design method and quantitative performance evaluation are necessary.

This paper will establish a key performance indicators model for the LEO plane array rotary-scan imaging system, including swath, resolution, and overlap rate between frames. Accordingly, it will put forward a kind of system parameter optimization design method based on the linear weighting and Monte Carlo method, finish the system parameter designs under a series of altitudes, and use the grid point method to evaluate the system covering properties. This will provide a theoretical basis for the design and application of array rotating scanning space cameras.

## 2. Low Earth Orbit Plane Array Rotary Scan Camera

**Figure 1:**
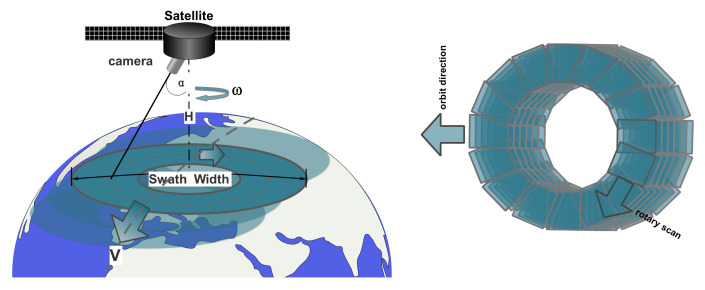
The imaging process with the LEO plane array rotary scan space camera. In which α is the camera’s tilt angle, *H* is the orbit height, ω is the rotating angular speed of the camera, and SW is the swath width.

The imaging principle of the LEO plane array rotary scan space camera is shown in Figure 1. The camera mainly comprises a plane array detector of high resolution, an optical system with a wide field and high resolution, and a one-dimensional rotating mechanism. The one-dimensional rotating axis is along the nadir direction, and the camera is fixed on the one-dimensional rotating mechanism with a tilt angle α. During the imaging process of the camera, the optical axis of the camera rotates at a certain angular speed ω. At the same time, the satellite runs along the orbit at a uniform speed V, making the optical axis of the camera form a scanning track in the shape of a solenoid on the ground. Many instantaneous fields of view form an annular strip field of view through the stitching of scanning images. Therefore, the low orbit plane array rotary scan camera can realize wide-area coverage imaging of the ground by tilt installation of the camera and rotating scanning of the one-dimensional rotating mechanism.

For this planar array rotary scan camera, in addition to realizing large-width and high-resolution imaging, it is necessary to avoid missing scans in the scanning process. This ensures that the observed, searched, and monitored targets will not be omitted in the scanning process. Therefore, only when the model of the swath, resolution, and scanning overlap rate of the plane array rotary scan camera is established can the parameter design and optimization of the system be carried out effectively.

### 2.1. Swath and Resolution Model

For the space camera, the scanning process increases the swath and reduces the resolution. The mutual restriction between the swath and resolution brings difficulties to the design of large-swath and high-resolution space cameras. At the same time, the influence of the curvature of the Earth’s surface on the resolution of the swath cannot be ignored for the imaging process of the large swath [17]. The simple traditional assumption that the ground is a plane is no longer applicable, and the curvature of the Earth’s surface must be considered. Therefore, an accurate model of swath and resolution is of significance for guiding the design of system parameters for the planar array rotary scan camera with a large swath area and high resolution.

**Figure 2:**
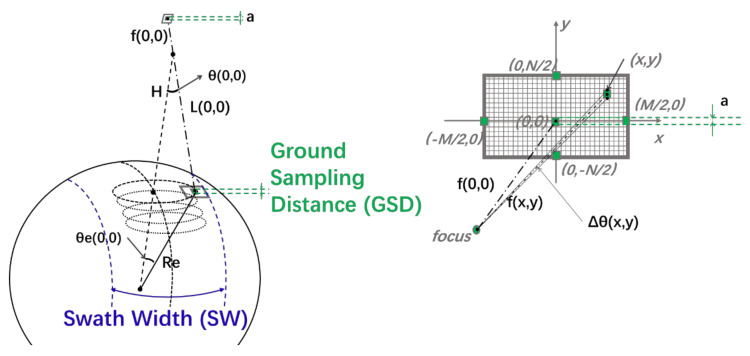
A diagram of the Swath Width (SW) and Ground Sampling Distance (GSD).

We assume that the Earth is a pure sphere and the scanning width SW is determined by the arc length perpendicular to the orbit running direction of the scanning area. The resolution, expressed by ground sampling distance GSD, is determined by the projection of the pixel at the center of the camera’s viewing axis, as shown in Figure 2. According to the geometric relationship, the scanning width and resolution can be obtained as follows:

(1) $$\mathrm{SW}=2R_{e}\cdot \theta_{e}\left(\frac{M}{2},0\right)$$

(2) $$\mathrm{GSD}=\frac{L\left(0,0\right)\cdot \Delta \theta \left(0,0\right)}{\cos\;\left(\theta \left(0,0\right)+\theta_{e}\left(0,0\right)\right)}$$

in which,

(3) $$\Delta \theta \left(x,y\right)=2arctan\frac{a}{2\cdot f\left(x,y\right)}$$

(4) $$f\left(x,y\right)=\sqrt{f^{2}+{\left(ax\right)}^{2}+{\left(ay\right)}^{2}}$$

(5) $$L\left(x,y\right)=\frac{Re\cdot sin\theta_{e}\left(x,y\right)}{sin\theta \left(x,y\right)}$$

(6) $$\theta_{e}\left(x,y\right)=\arcsin\left(\frac{\left(R_{e}+H\right)sin\theta \left(x,y\right)}{R_{e}}\right)-\theta \left(x,y\right)$$

(7) $$\theta \left(x,y\right)=\arccos\left(\cos\theta_{x}\left(x\right)\cos\theta_{y}\left(y\right)\right)$$

(8) $$\theta_{x}\left(x\right)=\alpha +\arctan\left(x\frac{a}{f}\right)$$

(9) $$\theta_{y}\left(y\right)=\arctan\left(y\frac{a}{f}\right)$$

As is shown in Figure 2, in Equation (1),$\;R_{e}$ is the radius of the Earth,$\;\theta_{e}\left(x,y\right)$ is the included angle of the subsatellite point’s plumb direction and ground object point’s plumb direction, and $\;\left(\frac{M}{2},0\right)$ is the mid-point of the outer edge of the detector. In Equation (2), $L\left(x,y\right)$ is the distance from the camera’s focus to the object point corresponding to the image point $\left(x,y\right)$, $\Delta \theta \left(x,y\right)$ is the angle resolution of the image point $\left(x,y\right)$, $\theta \left(x,y\right)\;$ is the included angle of the line of sight of the image point $\left(x,y\right)$ and nadir direction,$\;\left(0,0\right)$ is the center of the image detector. In Equation (3), a is the pixel size, and $f\left(x,y\right)$ is the distance from the camera’s focus to the image point $\left(x,y\right)$. $\left(x,y\right)$ is the image point of the object point on the image detector. In Equation (4), f is the focal length of the lens. In Equation (6), H is the orbital height. In Equation (7), $\theta_{x}\left(x\right)$ and $\theta_{y}\left(y\right)$ are, respectively, the projection of $\theta \left(x,y\right)$ in two orthogonal directions, as shown in Equations (8) and (9), where α is the installation angle of the camera and is equal to $\theta \left(0,0\right)$.

### 2.2. Overlap Rate

When the camera keeps scanning, there should be an overlap between the acquisition areas of two adjacent frames to avoid blind spots. The size of the overlap area will affect the target acquisition probability. If the overlap area is small, the scanning time is short, and the probability of target acquisition is low. On the contrary, the large overlap between two adjacent acquisition areas increases the probability of target acquisition but may increase scanning time [18]. Therefore, it is also necessary to establish a scan overlap rate model to ensure an appropriate overlap rate between adjacent frames.

**Figure 3:**
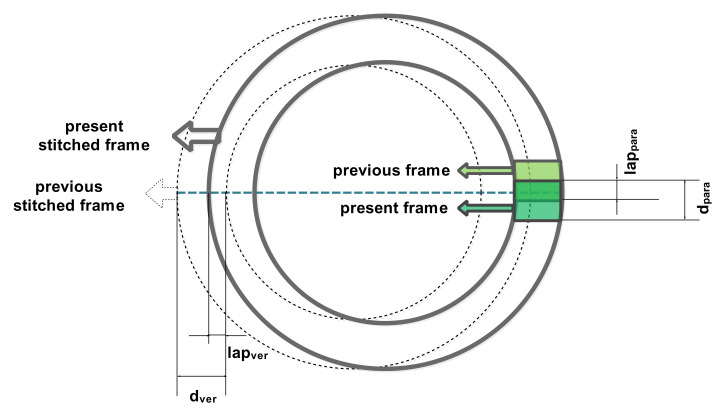
A diagram of the overlap between frames.

There are two kinds of overlap conditions for regional overlap in imaging, as shown in Figure 3. The first is the overlap between two adjacent “stitching frames” stitched by frames captured in the scan period. The overlap rate between stitched frames is *K*, as shown in Equation (10). When *K* is not less than zero, there is no leakage between adjacent circular stitching frames.

The second is the overlap between two adjacent instantaneous frames. The overlap rate between the instantaneous frames is η, as shown in Equation (11). When *η* is not less than zero, there is no missed area between adjacent instantaneous frames.

(10) $$K=\frac{lap_{ver}}{d_{ver}}$$

(11) $$\eta =\frac{lap_{para}}{d_{para}}$$

where $d_{ver}$ is the edge length in the vertical scanning direction of the coverage area, and $d_{para}$ is the edge length along the scanning direction of the coverage area. $lap_{ver}$ is the overlap length between the two adjacent splicing frames perpendicular to the scanning direction, and $lap_{para}$ is the overlap length between the adjacent two instantaneous frames parallel to the scanning direction, as shown in Equations (12)–(15).

(12) $$lap_{ver}=d_{ver}-V_{orbit}\cdot \frac{2\pi }{\omega_{scan}}$$

(13) $$d_{ver}=R_{e}\cdot \theta_{e}\left(\frac{M}{2},0\right)-R_{e}\cdot \theta_{e}\left(-\frac{M}{2},0\right)$$

(14) $$lap_{para}=d_{para}-\omega_{scan}\cdot L\left(0,0\right)\cdot sin\theta \left(0,0\right)\cdot \frac{1}{ftps}$$

(15) $$d_{para}=2R_{d}\cdot \theta_{d}$$

where $V_{orbit}$ is the orbit velocity, $\omega_{scan}$ is the scanning angular velocity of the rotating scanning, $V_{scan}$ is the scanning line velocity of the rotating scanning, and ftps is the frame frequency of the detector. $R_{d}\;$ and $\theta_{d}$ are the radius and radian of the tangential circle corresponding to the half field of view along the scanning direction passing through the center of the instantaneous field of view, as shown in Equations (16) and (17). $\left(\frac{M}{2},0\right)$ is the mid-point of the outer edge of the detector, $\left(-\frac{M}{2},0\right)$ is the mid-point of the inner edge of the detector, and N is the pixel number of the detector along the scanning direction, as shown in Figure 2.

(16) $$R_{d}=R_{e}\cdot cos\left(\arcsin\left(\frac{\left(Re+H\right)\cdot \sin\theta_{x}\left(0\right)}{Re}\right)\right)$$

(17) $$\theta_{d}=arcsin\frac{sin\theta_{y}\left(\frac{N}{2}\right)\left[R_{d}+L\left(0,0\right)\right]}{R_{d}}-\theta_{y}\left(\frac{N}{2}\right)$$

## 3. System Parameter Design Process

**Figure 4:**
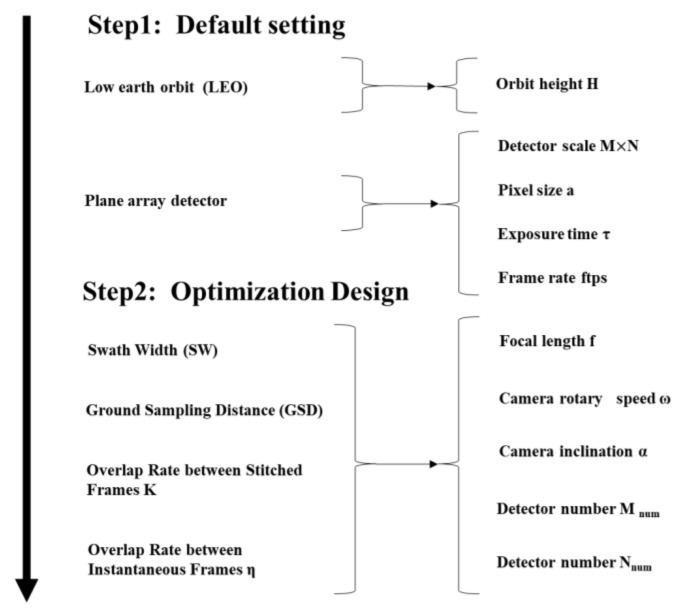
Diagram of the design process.

The design process of the array rotating scanning imaging system is shown in Figure 4. The first step is to select a kind of plane array detector with small pixel size and large detector scale and pick an orbit height in the low earth orbit range. The second step is to design and optimize the main overall system parameters of the plane array rotating scanning system according to the swath, resolution, and overlap rate model established above. The main system parameters for the lens subsystem, detector subsystem, and platform subsystem are all deduced in two steps.

### 3.1. Optimization Design Modeling

In the whole design process, the optimization design is the key problem. This will be used to deduce the camera installation inclination α, rotating mechanism speed $\omega_{scan}$, optical system focal length f, and detector patch number Mnum,Nnum. The optimization design problem can be described as follows:

Assume that the system width is not less than ${\mathrm{SW}}_{0}$ and the resolution is not less than ${\mathrm{GSD}}_{0}$ and there is no missing area in the scanning process. According to the task requirements, the constraint range of the overlap rate *K* and *η*, swath SW, and resolution GSD is proposed, as is shown in Equation (18).

(18) $$constraint1:\left\{\begin{array}{c} \mathrm{SW}\left(f,\omega_{scan},\alpha ,Mnum,Nnum\right)\geq {\mathrm{SW}}_{0};\\ \mathrm{GSD}\left(f,\omega_{scan},\alpha ,Mnum,Nnum\right)\leq {\mathrm{GSD}}_{0};\\ K\left(f,\omega_{scan},\alpha ,Mnum,Nnum\right)\leq K_{0}\\ \eta \left(f,\omega_{scan},\alpha ,Mnum,Nnum\right)\leq \eta_{0}; \end{array}\right.$$

Based on the design experience of the existing system, the parameters of the above systems are constrained: the camera installation angle α is constrained by the limit field of view angle, the speed $\omega_{scan}$ is restricted according to the stability requirements of the rotating mechanism, and the splicing length is restricted within $length_{max}$ according to the detector’s splicing ability. Focal length *f* is restricted within $f_{max}$, according to the existing lens design capabilities [19], as is shown in Equation (19).

(19) $$constraint2:\left\{\begin{array}{c} f_{min}\leq f\leq f_{max};\\ {\omega_{scan}}_{min}\leq \omega_{scan}\leq {\omega_{scan}}_{max}\\ \alpha_{min}\leq \alpha \leq \alpha_{max}\\ Mnum\cdot a\leq length_{max}\\ Nnum\cdot a\leq length_{max} \end{array}\right.$$

Two design objects are focusing on performance and design difficulty, respectively. The width is as large as possible for the objective performance function, and the resolution is as small as possible. Thus, two objective performance functions are obtained, as Equation (20) shows Equation (21). For the design difficulty objective function, the design difficulty mainly includes the optical system focal length, rotation speed, and the number of detector system splices—generally, the shorter the focal length, the more compact the system volume structure. The smaller the rotating speed, the smaller the motor power. The smaller the number of detectors, the smaller the difficulty of stitching. Thus, three design difficulty objective functions are obtained as shown in Equations (22)–(24).

(20) $${Object}_{p1}=\max\left(\mathrm{SW}\right)$$

(21) $${Object}_{p2}=\min\left(\mathrm{GSD}\right)$$

(22) $${Object}_{d1}=\min\left(f\right)$$

(23) $${Object}_{d2}=\min\left(\omega_{scan}\right)$$

(24) $${Object}_{d3}=\min\left(Mnum\cdot Nnum\right)$$

Therefore, according to the performance requirements of the width and resolution no-leakage scanning, under the limitations of the existing system design level and feasibility, the key system parameters are designed to achieve the minimum design difficulty and the best performance parameters.

From the above analysis, it can be seen that the design process of the low-orbit array scanning camera is to solve the parameters of $\left(f,\omega_{scan},\alpha ,Mnum,Nnum\right)$. In this planning problem, the objective functions are multiple objectives, and the constraint conditions are nonlinear functions with trigonometric functions. At the same time, the decision variables Mnum and Nnum must be integers. Therefore, this problem is a mixed-integer nonlinear programming problem with multiple optimization objectives and is a complex planning problem.

### 3.2. Optimization Design Method

In the optimization process, there are two key methods. The first method is to transform the multi-objective function into a single objective function via the linear weighting method. For the multi-objective optimization problem, the linear weighted summation method can simplify the multi-objective optimization problem into a single-objective problem by assigning the multiple objectives with appropriate weight coefficients according to their importance and building a new objective function to solve the optimization problem. As shown in Equations (20)–(24), the five objective functions can be divided into two kinds: the performance objective function term and the design difficulty objective function term. For the performance objective function term, the weight of w1 is assigned to the width and average resolution. The remaining weight of w2 is given to the focal length, rotational speed, and the number of detectors on average. Therefore, the multi-objective function is simplified to a single-objective function, and the new objective function is shown in Equation (25). Considering that the constraint conditions have limited the performance indexes, the weight of the performance objective function is set to w1=0.2, and the weight of the difficulty objective function is set to w2=0.8.

(25) $$Object=w1\cdot \left(Object_{p1}+Object_{p2}\right)/2+w2\cdot \left(Object_{d1}+Object_{d2}+Object_{d3}\right)/3$$

The second method is to solve the mixed nonlinear optimization problem via the Monte Carlo method. This method is an effective solution for the mixed-integer nonlinear programming problem. Therefore, the parameter design method is determined as multi-objective linear-weighted Monte Carlo optimization. For the Monte Carlo optimization method, the convergence criterion or the termination condition of operation of its optimization should be reasonably given to obtain the optimal parameter model and save calculation time. Here, the convergence criterion is designed as follows: (1) Complete the maximum number of feasible solutions, the operation terminates; (2) complete the maximum number of operations, the operation terminates. When any one of the two criteria is satisfied, the operation can be terminated.

Thus, the process steps of parameter design are as follows: Firstly, a set of parameters $x=\left(f,\omega_{scan},\alpha ,Mnum,Nnum\right)$ is randomly generated according to a uniform distribution. Then, whether the constraint in Equations (23) and (24) is satisfied or not is checked. If the solution is feasible, the objective function in Equation (25) of the current system parameter *x* is calculated. If it is better than the current optimal solution object_best, *x* is updated to the optimal solution; otherwise, the next solution is continued. After, determine whether the current convergence conditions are met. If so, terminate the operation; otherwise, continue the circle. The specific process is shown in the following pseudo-code as Algorithm 1.
**Algorithm 1: System parameter design algorithm**1: **Begin** 2: Initialize calculation number: ***i* = 0**; 3: Initialize feasible solution number: ***k* = 0**; 4: **Input** maximum calculation number: ***i_max***; 5: **Input** maximum feasible solution number: ***k_max***; 6: **while**   **(*i < i_max) and (k < k_max)* do** 7:   Update the calculation number ***i = i +* 1**; 8:   Generate a set of system parameters at random: ***x(i)* = (*f, w, alpha, Mnum, Nnum*)**; 9:   **if *(Constraint1*** in Equation (18)**) *and (Constraint2*** in Equation (19)***) == TRUE* then** 10:    Save ***x(i)*** as a feasible solution; 11:     update the feasible number: ***k = k + 1***; 12:     **if *object(i) ≤ object_best* then** 13:        Update the best solution: ***x_best = x(i), object_best = object(i)***; 14:     **end** 15:   **end** 16:   **Output** The best solution ***x_best***; 17: **end** 18: **End**

## 4. Example and Evaluation

### 4.1. Design Example

Taking “SW ≥ 1000 km and GSD ≤ 1 m” and “no-leakage scanning” as the design goal, the plane array rotary scan imaging system is designed in the low altitude range. According to the design process shown in Figure 4, the first step is to determine orbit height and pick the detector and the second step is the optimization design.

In the first step, orbital height is 200–1000 km as the orbit is set at low earth orbit. According to the principle of “small pixel size, large array scale,” the detector CMOS-GSENSE5130 is selected. The pixel size is 4.5 μm, the single-chip detector size is 5092 × 3021, and the detector frame frequency is 67 Hz.

In the second step, the system parameters are designed according to the Monte Carlo method shown in Algorithm 1. Specifically, the maximum number of operations is *i*_max = 1,000,000 and the maximum number of feasible solutions is *k*_max = 1000. The optimization constraint is “SW ≥ 1000 km and GSD ≤ 1 m” and “*K* ≥ 0 and *η* ≥ 0.” The optimization goal is to acquire the minimum objective function, as is shown in Equation (25).

Through the two steps, optimization design results are simulated, shown in Table 1 as below.

It is shown in Table 1 that the optimal solution of different orbital heights in the range of 200–1000 km can achieve the design goal of “SW ≥ 1000 km and GSD ≤ 1 m,” and there is no leakage during scanning. The objective functions of the design results with track heights of 500 km, 600 km, and 800 km are the smallest, reaching 0.46. Among the three optimal results, the design result at 500 km has the lowest orbit height, which means that the energy required for the satellite launch is the smallest and has the shortest focal length, which means that the design difficulty of the optical system is the smallest. The result at 600 km has the highest resolution. The result at 800 km has the lowest rotational speed for the rotating mechanism and the smallest inclination angle of the camera, which means the design difficulty of the one-dimensional rotating mechanism is the smallest and has the greatest width. The design results under three orbital heights have their advantages and can be further selected according to the difference of tasks.

### 4.2. Performance Evaluation

Based on the optimal design result of 500 km, we evaluate the target detection performance of the array rotating scanning system. Assume that the target detection task is searching and rescuing ships and planes wrecked over the South China Sea. For target detection tasks, coverage characteristics are particularly important. For static targets, the camera needs to cover a wide range as fast as possible. For moving targets, the camera also needs longer observation periods at a higher frequency to prevent omission.

Therefore, the main evaluation indexes include the accumulated coverage rate, observation duration, and repeated observation frequency during the observation duration when the satellite passes through the target region. The simulation result is compared with the traditional push broom camera and whisk broom camera to evaluate the target-detection ability of the rotary-scan camera in search and rescue missions.

The orbital parameters are set as follows: the orbital height of the satellite is 500 km, the equatorial longitude of the ascending point is 105.4° E, the initial operating position angle is 10.6°, and the orbital inclination angle is 70.2°. The push broom and whisk broom camera system parameters are set to be the same as the evaluated rotary-scan camera: the optical and detector parameters are shown in the design results of 500 km orbital height in Table 1. The swing angle range and swing angular velocity of the scanning camera is set to be −43.24°~+43.24° and 65.61°/s, respectively.

The grid point method [20] is used to conduct numerical simulation analysis on the coverage of the target region, which turns the coverage performance evaluation problem into a numerable statistical analysis of the latitude-longitude grid points. For the target simulation region, the longitude ranges from 109° E to 118° E and the latitude ranges from 10° N to 35° N. The target simulation period is when the line of sight crosses through the South China Sea region. The grid-scale is selected as 0.05°. All the parameters are shown in Table 2.

**Figure 5:**
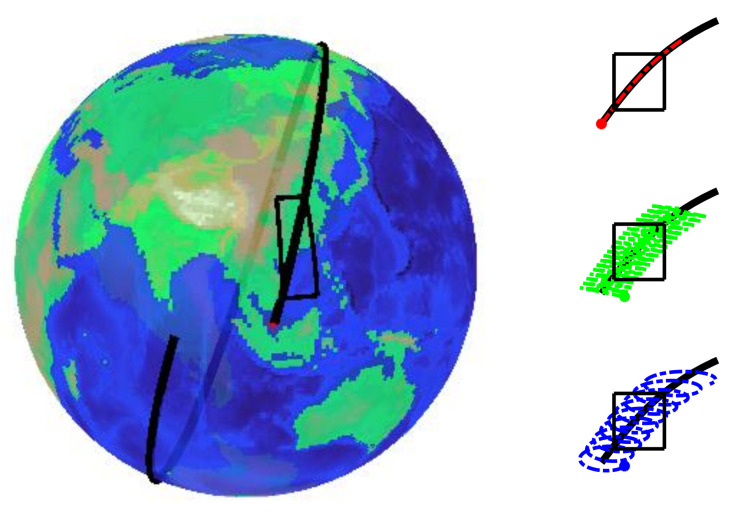
A diagram of the three scanning imaging modes.

The scanning coverage of the center of the line of sight of the three scanning cameras is shown in Figure 5. The line of sight for the push broom, whisk broom, and rotary scan is shown in red, green, and blue, respectively.

**Figure 6:**
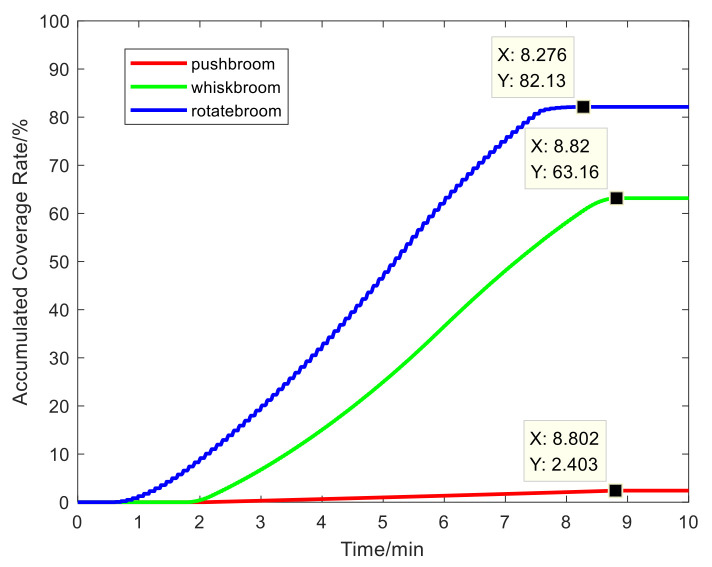
A diagram of the accumulated coverage rate.

The accumulated coverage rate simulation results of the three scanning imaging systems are shown in Figure 6. Within 8 min of satellite transit time, the maximum coverage rates of the observation area for the push broom, whisk broom, and rotary scan are 2.40%, 63.17%, and 82.13%, respectively. The accumulated coverage rates of the whisk broom and rotary scan are greatly improved compared with the push broom, especially in rotary scan imaging mode. Moreover, it takes 8.8 min, 8.82 min, and 8.2 min for the push broom, whisk broom, and rotary scan to reach the maximum coverage rate, respectively. The push broom and whisk broom times are essentially the same, while the time for rotary scanning is 30 s less.

Obviously, under the same system parameters, rotary-scan imaging can achieve a larger coverage rate in the shortest time among the three scanning imaging modes. This will greatly improve the efficiency of targeted search and rescue.

**Figure 7:**
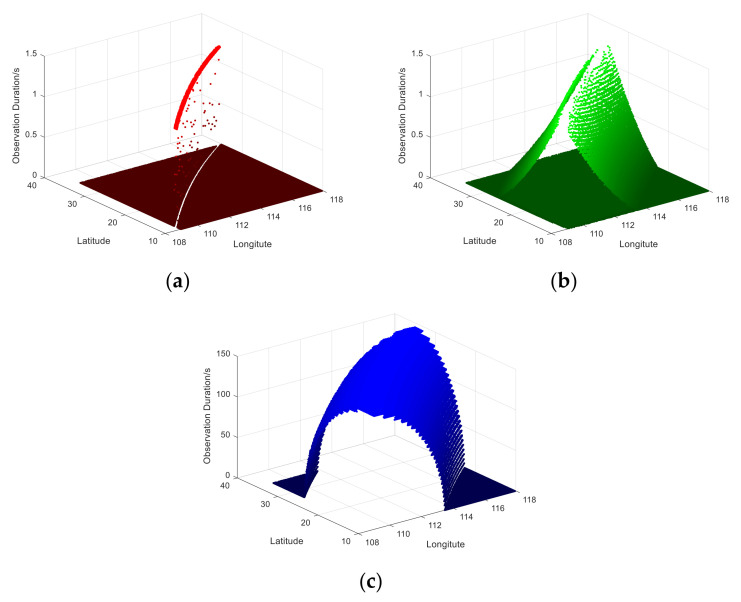
A diagram of the observation time duration. (**a**) Push broom; (**b**) Whisk broom; (**c**) Rotary scan.

The observation duration is from the first moment to the last in the observed region. The simulation results of the observation time of the three scanning imaging systems are shown in Figure 7. During the transit time of the satellite for the observation area, the average observation times of the push broom, whisk broom, and rotary scan are 1.19 s, 0.17 s, and 118 s, respectively. The maximum observation times are 1.22 s, 1.22 s, and 145.73 s, respectively. The observation duration of the rotary scan camera is longer than that of the traditional push broom and whisk broom cameras.

**Figure 8:**
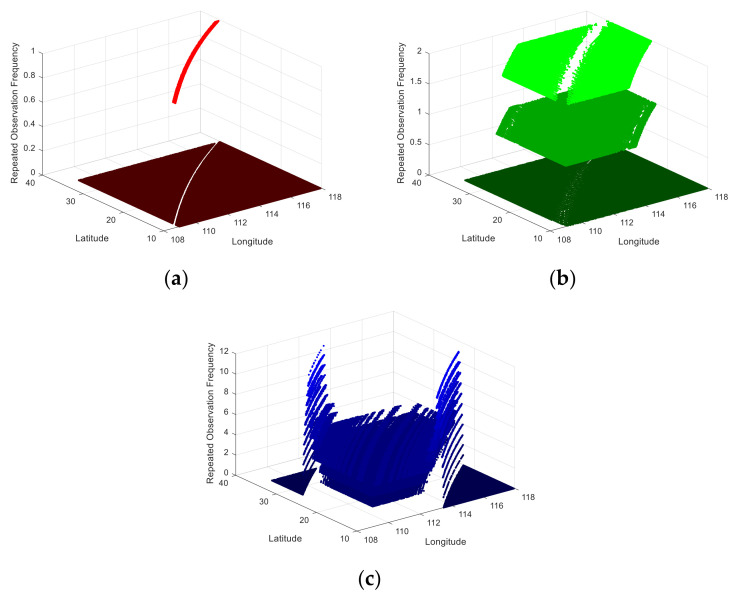
A diagram of the frequency of repeated observation. (**a**) Push broom; (**b**) Whisk broom; (**c**) Rotary scan.

The repeated observation frequency during the observation duration is shown in Figure 8. During the transit time of the satellite, the average number of repeated observations in the observation area by the push broom, whisk broom, and rotary scan is 1, 1.32, and 3.26, respectively, and the maximum repeated observation frequency is 1, 2, and 12, respectively. The traditional push broom cannot observe repeatedly, and the optical machine scanning significantly improves the repeated observation performance of the imaging system. With the same system parameters, the repeated observation frequency of the rotating scanning mode is enhanced by 200% compared with that of the traditional push broom.

Longer observation time and higher repeated observation frequency enable the rotary scan to provide more opportunities to find the rescue target, thereby greatly improving the targeted search and rescue success rate.

Apparently, for the same orbit and camera parameters and the same selected observation and observation areas, the rotary scan camera has obvious advantages over the traditional push broom and whisk broom methods. The first advantage is the higher coverage rate over a shorter time. The second advantage is higher repeated observation frequency over a longer observation duration. These two advantages can greatly improve the efficiency and possibility of search and rescue missions. This means that the low-orbit array rotary scan camera has great potential to achieve efficiently, targeted search and rescue tasks over wide areas.

## 5. Conclusions

In this paper, a low-orbit plane array rotary scan camera is brought into focus. Based on the swath, resolution, and overlap rate modeling, a system parameter optimization design method combining Linear Weighting and the Monte Carlo method is proposed. This method can quickly and easily design the initial system parameters, and the designed system can provide large width, high resolution, and non-leakage scanning imaging. The gridpoint method’s evaluation shows that the designed rotary-scan camera has the advantages of “high coverage rate via fast scanning” and “high repeated observation frequency over a long duration of observation”, compared with the traditional push broom and whisk broom. The design method and evaluation results of system parameters proposed in this paper lay a theoretical foundation for the low-orbit array rotary scan camera design. They provide important technical support for the engineering application, hopefully improving the target observation ability in targeted search-and-rescue tasks over wide areas.

## Figures and Tables

**Table 1:** Design results of system parameters at a typical low orbital altitude.

No.	*H*	*f*	*w*	alpha	*M_num_*	*N_num_*	SW	GSD	*K*	ETA	*Object*
1	200	7.78	62.13	66.32	10	10	1,054,346	0.96	0.11	0.14	0.61
2	300	5.63	98.15	56.7	7	12	1,005,317	0.96	0.2	0.25	0.56
3	400	5.15	80.66	49.88	8	8	1,037,295	0.98	0.19	0.23	0.50
**4**	**500**	**4.78**	**65.61**	**43.24**	**9**	**6**	**1,022,767**	**0.99**	**0.14**	**0.30**	**0.46**
**5**	**600**	**5.21**	**61.08**	**39.46**	**10**	**5**	**1,072,620**	**0.97**	**0.17**	**0.21**	**0.46**
6	700	6.07	64.75	35.92	10	5	1,091,257	0.87	0.15	0.10	0.47
**7**	**800**	**5.93**	**54.11**	**34.7**	**10**	**5**	**1,196,224**	**0.99**	**0.12**	**0.29**	**0.46**
8	900	6.33	69.87	32.79	8	8	1,238,651	1.00	0.17	0.42	0.51
9	1000	7.34	67.64	28.64	9	6	1,159,952	0.86	0.14	0.23	0.51

**Table 2:** Design results of the system parameters at a typical low orbital altitude.

Parameter	Value
Target simulation region:	Longitude: [109° E, 118° E];
The South China Sea region.	Latitude: [10° N, 35° N]
Grid-scale	[0.05, 0.05]
Orbit height	500 km
Equatorial longitude of the ascending point	105.4° E
Initial operating position angle	10.6°
Orbit inclination	70.2°
Focal length	4.78 m
Detector type	CMOS-GSENSE5130
Pixel size a	4.5 μm
Detector Scale M*N	5092 × 3021
Frame frequency	67Hz
Splicing Number M_num_*N_num_	9 × 6
Swing angle alpha	−43.24°~+43.24°
Swing angular velocity	65.61°/s
